# Haemosporidian Infection Risk Variation Across an Urban Gradient in a Songbird

**DOI:** 10.1002/ece3.71770

**Published:** 2025-07-09

**Authors:** Wilmer Stanley Amaya‐Mejia, Lillian Ma, Sara Freimuth, Ravinder N. M. Sehgal, Pamela Yeh

**Affiliations:** ^1^ Department of Ecology and Evolutionary Biology, UCLA Los Angeles California USA; ^2^ Department of Biology, SFSU San Francisco California USA; ^3^ Santa Fe Institute Santa Fe New Mexico USA

**Keywords:** avian infectious disease ecology, dark‐eyed juncos, urban ecology, vector ecology

## Abstract

Urbanization is a significant source of inter‐ and intra‐city environmental variation and is associated with declining avian population sizes, with a shift towards more homogeneous communities that consist of large populations of the same species. However, whether this shift extends to urban disease ecology and related parasite communities requires further examination. By comparing the diversity of two related parasite genera (largely host‐generalist *Plasmodium* and largely host‐specialist *Haemoproteus)* and infection status of dark‐eyed juncos ( *Junco hyemalis* ) across an urbanization gradient in California, we can determine how broad urban‐associated land use changes and localized habitat composition correlate with pathogen communities. Additionally, by examining vector abundance responses, we can begin to assess broader impacts on urban disease transmission and ecology. We report fewer birds were infected with *Haemoproteus* in urban habitats, with a larger presence of host‐generalist lineages, suggesting urbanization increases homogenization of host‐specialist pathogens. Unsurprisingly, the largely host‐generalist pathogen, *Plasmodium,* showed no correlation with urbanization, but infections increased with rainfall. Local habitat characteristics had limited effects on *Plasmodium* infection status, but biotic characteristics, including wing chord length and human presence, were associated with *Plasmodium* infections. Lastly, *Culex tarsalis,* an important vector for *Plasmodium* and zoonotic pathogens, was the only vector to also increase in abundance in response to rainfall. Our results show that broad land use changes associated with urbanization decrease avian parasite biodiversity and highlight localized abiotic and biotic habitat characteristics that may reduce infection prevalence.

## Graphical abstract

Avian haemosporidian community composition of a songbird was assessed across an urbanization gradient. Broad urban‐associated land use changes corresponded with reduced host‐specialist parasites and reduced prevalence of avian parasites. Select local habitat characteristics and vector abundance may present additional risks for infection prevalence.

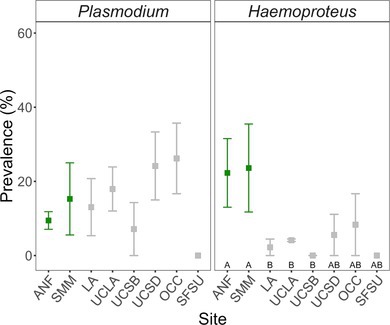

## 1 Introduction

Multiscale human‐induced habitat changes, mainly through urbanization, impact established ecological and evolutionary systems (McKinney 2006; Grimm et al. 2008; Rosenberg et al. 2019). Urban spaces are characterized by increased impermeable surface coverage, reduced vegetation coverage, increased human presence, elevated temperatures, and increased pollution (Oke 1973; Grimm et al. 2008; Ziter et al. 2019; Miles et al. 2019). Cities with these attributes have been associated with decreased native biodiversity, increased biological homogenization, and are expected to contribute to declining wildlife population sizes (McKinney 2006; Aronson et al. 2014; Dirzo et al. 2014; Rosenberg et al. 2019). However, there are instances of urban‐adapted species (Neate‐Clegg et al. 2023), and many pathogens, in particular, are reported to increase in cities (reviewed by Bradley and Altizer 2007). Our current understanding of the effects of urbanization, as it relates to disease ecology, suggests a complex, potentially multiscale, impact that warrants further study.

Prominent inter‐and intra‐city variation is the result of broad and local environmental characteristics (Wolch et al. 2014; Beninde et al. 2015; Vahmani and Ban‐Weiss 2016; LA Sanitation and Environment 2018; Nardone et al. 2020). This variation can have large‐scale drivers, including the conditions associated with cities (Grimm et al. 2008; Ziter et al. 2019; Miles et al. 2019), but can also include differences in climatic patterns (Oke 1973; Vahmani and Ban‐Weiss 2016; Mokhtari et al. 2022; Harrison et al. 2024). Large‐scale changes can also obscure important small‐scale variability. Within cities, greenspaces found in parks provide structural complexity, which can offer climate refugia and allow for the retention of local biodiversity (Assadi et al. 2015; Threfall et al. 2016; Blinkova and Shupova 2017; Magle et al. 2019). When studying urban disease ecology, it may be necessary to account for both the broad intercity variability and localized intracity habitat variability to determine if these characteristics contribute to the complex patterns previously observed (Bradley and Altizer 2007).

Avian haemosporidians are protozoan obligate parasites, the causative agents of avian diseases including malaria, and are an important model system for understanding and predicting the effects of environmental change on disease ecology and evolution, with implications for wildlife and human health (Atkinson and Van Riper III 1991; Martinsen et al. 2008; Delgado and French 2012; Clark et al. 2014; Guo et al. 2019). The prevalence of avian haemosporidian pathogens has previously been associated with habitat conditions in naturally occurring ecosystems (Beadell et al. 2004; Bonneaud et al. 2009; Sebaio et al. 2010; Sehgal et al. 2011; Gonzalez‐Quevedo et al. 2014; Carbó‐Ramírez et al. 2017; Ferraguti et al. 2018; Tchoumbou et al. 2020; Menzies et al. 2021; De Angeli Dutra et al. 2023). Although there are many landscape traits that can affect the infection prevalence of avian haemosporidians, vegetation characteristics (Bonneaud et al. 2009; Carbó‐Ramírez et al. 2017) and water availability (Gonzalez‐Quevedo et al. 2014; De Angeli Dutra et al. 2023) were frequently positively associated with an increase in infection prevalence. While urbanization directly impacts local vegetation (Blinkova and Shupova 2017) and could have significant downstream effects on urban pathogen communities (Bonneaud et al. 2009, Carbó‐Ramírez et al. 2017), changes in water availability, including precipitation, may prove equally as impactful.

Avian haemosporidians encompass several genera of parasites, including *Plasmodium* and *Haemoproteus* (Valkiūnas 2005; Hellgren et al. 2015). These two genera are phylogenetically related, but mosquitoes transmit *Plasmodium* spp. and are largely found to infect various unrelated avian host species. In contrast, *Haemoproteus* spp. are usually transmitted by biting midges and are more likely to be host specialists, although some species and lineages are capable of infecting a range of host species (Beadell et al. 2004; Svensson‐Coelho et al. 2013; Martínez‐Renau et al. 2022). Many studies have reported that the prevalence of generalist parasites in hosts, such as *Plasmodium* spp., more readily responds to environmental conditions, especially water availability. In contrast, infection prevalence and diversity of host‐specialist parasites, such as *Haemoproteus* spp., primarily increase in response to greater host diversity (Santiago‐Alarcon et al. 2013; Loiseau et al. 2017; Abella‐Medrano et al. 2018; Amaya‐Mejia et al. 2022). This connection between *Haemoproteus* infection prevalence and host communities has been attributed to energy availability, specifically how energy input versus transfer can increase the available energy and subsequently increase host community diversity (Darío Hernandes Córdoba et al. 2024), but this may decline in urban habitats with reduced green spaces. These studies suggest that the risk of a *Plasmodium* or *Haemoproteus* infection will vary between the two genera of parasites as a result of different life histories and, indeed, differences in their respective vectors.

Population size of *Culex* mosquitoes, an important vector for *Plasmodium* spp. (Carlson et al. 2015), can vary widely along precipitation and urbanization gradients. For example, while the abundance of *Cx. tritaeniorhynchus*, *Cx. gelidus* (Olson et al. 1983), and *Cx*. *quinquefasciatus* (Valdez et al. 2017) generally increases with rainfall, excessive rainfall can have detrimental effects (Olson et al. 1983; Valdez et al. 2017). In response to urbanization, some species of mosquitoes, such as *Cx. restuans*, have lower abundance in urban habitats (Arsenault‐Benoit and Fritz 2023), while others increase (Farajollahi et al. 2011). Therefore, to better understand how urbanization affects vector‐borne pathogen transmission, information on vector ecology is also needed.

Dark‐eyed juncos ( *Junco hyemalis* ), specifically the Oregon juncos (Friis, Atwell, et al. 2022), have established populations across an urban gradient in central and southern California, presenting a valuable study system for urban disease ecology (Yeh and Price 2004; Atwell et al. 2014; Friis, Vizueta, et al. 2022; Diamant and Yeh 2024). While the degree of admixture between populations is unknown, there are documented residential urban populations and nearby migratory non‐urban populations (Yeh and Price 2004; Bressler et al. 2020; Diamant and Yeh 2024). Based on field observations, males are generally territorial with no instances of an urban junco moving to a non‐urban habitat, or vice versa, within a breeding season documented. Juncos have also previously been studied to assess junco‐avian haemosporidian disease systems both within and across populations reporting several different species and lineages of the genera *Plasmodium* and *Haemoproteus* (Deviche et al. 2001; Bears 2004; Hanauer 2017; Slowinski et al. 2018; Becker et al. 2019; Martínez‐Renau et al. 2022; Talbott and Ketterson 2023).

In this study, we compared how multi‐scale ecological characteristics correspond with *Plasmodium* and *Haemoproteus* infectious status of dark‐eyed juncos across an urbanization gradient. We compared infection status at the genus level and presented habitat characteristics which best explained variation across habitats. For *Plasmodium*, we expect (1) water availability, measured through precipitation, to positively correlate with infection status. As infections require the presence of competent pathogen vectors, (2) mosquitoes that can serve as vectors for *Plasmodium* should also increase with precipitation. Host communities are not directly observed in this study, but urbanization was previously found to decrease abundance and increase homogenization. As a result, we expect that (3) there are fewer instances of host‐specialist parasite infections, *Haemoproteus*, in urban habitats. We do not have specific a priori hypotheses for local scale associations; instead, we focus on describing which habitat and vegetation characteristics are the most predictive of infection status. The collective results highlight both broad environmental variables that correspond with the infection prevalence of avian haemosporidian parasites across cities while offering potential local scale variables for future consideration.

## 2 Materials and Methods

### 2.1 Field Sampling

Bird capture was conducted during three consecutive breeding seasons (January–August) from 2021 to 2023. The study sites spanned four major metropolitan areas (Los Angeles, San Diego, Santa Barbara, and San Francisco, CA). These included two non‐urban sites (Angeles Nation Forest, ANF and Santa Monica Mountains, SMM) where migratory populations reside during the breeding season (March–August) and six urban sites (University of California, Los Angeles campus, UCLA; University of California, San Diego campus, UCSD; University of California, Santa Barbara, UCSB; Occidental College campus, OCC; San Francisco State University campus, SFSU; and parks throughout Los Angeles, LA) where breeding populations remain residential year round.

At each site, juncos were captured by targeted mist netting. Trapping efforts included audio lures recorded from juncos in Los Angeles. All captured individuals, including males, females, and juveniles, were used in this analysis. Individuals were banded with metal federal aluminum bands and three plastic color bands to create unique band combinations for behavioral studies performed in tandem with this study. Body morphometrics were recorded, including weight, wing chord length, tail length, tarsus length, bill width, depth, and length. Age was determined based on plumage characteristics, and sex by combining plumage and brood patches or cloacal protuberances (Pyle et al. 1997). Blood samples, < 1% of the total mass with an average volume of 50 μL, were collected from individuals via brachial venipuncture using a 30G needle following sterilization of the puncture site with alcohol pads. Blood was collected via heparinized capillary tubes. For each bird, two blood smears were prepared per individual, fixed in the field and stained with Giemsa‐Wright, and whole blood was then stored in 500uL of Queen's lysis buffer (10 mM Tris pH 8.0, 100 mM ethylenediaminetetraacetic acid [EDTA], 2% sodium dodecyl sulfate [SDS]) at room temperature until extractions (Valkiūnas 2005; Owen 2011). Slides were used to confirm presence/absence of infections but were not used to calculate parasitemia in our current study. Samples were collected from recaptured individuals if the time between capture dates exceeded 2 weeks to account for novel infection acquisition.

### 2.2 Abiotic Environmental Assessment

Habitats were characterized by local vegetation surveys and remote sensing data. For both, values were obtained within a 50 m radius with the capture site set as the centroid. This equates to a reasonable approximation of the space used by small territorial passerines, such as juncos (Chandler et al. 1994; Blair 2001; Wood et al. 2016; Sottas et al. 2020).

Local vegetation was assessed between 2022 and 2023 with a modified Rapid Assessment Protocol (CNPS‐RAP) developed by the California Native Plant Society and the California Department of Fish and Wildlife (California Native Plant Society 2022). The main modification involved using the junco capture location as the centroid, regardless of whether it fell within the CNPS‐RAP definition of a vegetation stand. Additionally, the number of trash cans and tables within the assessment plot was counted as a proxy for anthropogenic waste availability (Mazué et al. 2022).

Broader habitat conditions included the Built‐Up Index (BU) as a metric for urbanization and monthly precipitation for water availability. The BU index was calculated based on the difference between the normalized difference built‐up index (NDBI) and the normalized difference vegetation index (NDVI) (He et al. 2010). The raster files for both the NDBI and the NDVI were from the U.S. Geological Survey Earth Resources Observation and Science (EROS) Science Processing Architecture (ESPA) Collection 2 Level 2 Landsat Surface Reflectance‐Derived Spectral Indices and had a resolution of 30 m. The monthly precipitation values were obtained from the PRISM Climate Group via *prism* 0.2.0 pack in R. The total rainfall for the month of the capture date was obtained.

### 2.3 Laboratory Analysis

DNA was extracted from whole blood lysis solution via a Qiagen DNeasy Blood and Tissue Extraction Kit (San Diego, CA, USA) or a Wizard SV Blood and Tissue Extraction Kit (Madison, WI, USA) following the manufacturer's instructions, which have been found to have comparable DNA extraction yields when screening for *Plasmodium* (Mann et al. 2015). A total of 10 μL of DNA was then used to screen for the presence of *Haemoproteus*/*Plasmodium* via the previously described nested PCR protocol (Waldenström et al. 2004). In brief, an initial 25‐μL reaction was performed with the primers HaemNF and HaemNR2, followed by HaemF and HaemR2 for nested PCR (Waldenström et al. 2004). All reactions were conducted via ThermoFisher DreamTaq MasterMix (Hanover Park, IL, USA). Positive samples were submitted for Sanger sequencing (Azenta US Inc., La Jolla, CA, USA). No coinfections were detected via chromatography; however, this is likely due to PCR bias, which preferentially amplifies *Haemoproteus* over *Plasmodium* (Ciloglu et al. 2019).

Sanger sequences were used to identify parasite genera and lineages. All sequences could be identified to the genus level. However, as different lineages can vary by as little as 4 bp (Bensch et al. 2009), only sequences with > 80% high‐quality reads were used to assign lineages to account for sequencing errors. All sequences were initially aligned via the MUSCLE alignment feature available in Geneious Prime 2025.1.2, with all sequences that exceeded 1% (4 bp) dissimilarity classified as a unique lineage (Bensch et al. 2009). Each unique lineage was compared via BLAST with sequences available in the GenBank and MalAvi databases to determine if it was previously observed (Bensch et al. 2009).

Cladograms for all lineages in our study were prepared based on the lineages in our study and reference lineages taken from previous studies in California (Oakgrove et al. 2014; Carlson et al. 2015; Walther et al. 2016). We initially aligned all lineage sequences using the MUSCLE 5.1 (Edgar 2022) algorithm. The alignment was trimmed to remove non‐overlapping regions, resulting in a final 438 bp alignment length. This 438 bp alignment was used in the *phangorn* package in R to determine cladogram parameters. The final cladogram was prepared with MrBayes 3.2.6 (Huelsenbeck and Ronquist 2001) in Geneious Prime 2025.1.2 and used the GTR + I + Γ model with 1 cold and 2 hot Monte Carlo Markov chains that were sampled every 1000 generations over 1 million generations. Across all samples, we discarded 25% as burn‐ins and used the remaining to construct the majority consensus tree and to calculate posterior probabilities.

Host–parasite specificity was reported as S_TD_ and calculated via TAXOBIODIV2 (http://www.otago.ac.nz/parasitegroup/downloads.html). While other metrics of host–parasite specificity exist (Svensson‐Coelho et al. 2013), S_TD_ is a comparable and widely used method (Poulin and Mouillot 2005). The data on hosts were based on reported observations available from the MalAvi data for each parasite lineage. Host–parasite specificity was reported via S_TD_ values ranging from 1 to 4, with higher values suggesting more generalist parasites. Lineages that were unique from those previously published were given a default S_TD_ value of 1.

### 2.4 Statistical Analysis

The complete dataset was divided by taxa, describing the relative absence of *Plasmodium* or of *Haemoproteus* infections for all subsequent analyses unless otherwise noted. All the statistical analyses were performed in R 4.2.1 and RStudio 2023.09.1 (R Core Team 2021). The analysis focused on the following: (1) variation between sites, (2) differences in host–parasite specificity between habitat types, and (3) significance of the effects of precipitation and urbanization. Two separate analyses were run at the individual level for *Plasmodium*: (4) determination of which local habitat conditions were most directly correlated with infection status, which were finally used to (5) determine which specific habitat conditions are most predictive of acquiring a *Plasmodium* infection.

Initially, the mean infection prevalence of all eight sites (LA, OCC, SFSU, SMM, UCLA, UCSB, UCSD, and ANF) for the three sampled years was compared with a Fisher's exact test for multiple pairwise comparisons using the *RVAideMemoire* 0.9–83‐3 package with α = 0.05.

Sites represent a broad categorization which could obscure more nuanced environmental variation. To account for this, the remaining analyses were performed with BU index as an explanatory factor and Site was removed to avoid overfitting our models. Similarly, the infection status of birds was expected to show seasonal variation, both within and between years. While variation within a year could only be measured based on ordinal dates, we chose to only capture variation between years using differences in precipitation as this was more biologically relevant (Olson et al. 1983; Gonzalez‐Quevedo et al. 2014; Valdez et al. 2017; De Angeli Dutra et al. 2023) and prevented overfitting of our models.

Host–parasite specificity for each lineage (*Plasmodium* lineages = 7, *n* = 78; *Haemoproteus* lineages = 6, *n* = 29) in our study was compared across sites by running a generalized linear model (GLM) with inverse gamma distribution. Inverse gamma distribution was selected by reviewing the model residuals. The S_TD_ of each infected individual was the response variable, and the corresponding BU index was set as a fixed effect variable. Due to the small sample size of unique lineages, including random effects resulted in an overfit model and were therefore excluded from our results.

To determine how infection status varied in response to urbanization and water availability, we ran a series of binomial generalized linear mixed models (GLMM) using the *lme4* 1.134 package. Infection status for each individual was set as the response variable for all models. We had a null model in which only seasonal variation, based on month, was set as a random intercept. We did not include additional variables, specifically year or site, as these variables lead to an overfit model and month captures much of the seasonal variation. Two additional models were run with month as a random intercept and either cumulative rainfall or BU index values as a fixed effect. A full model, with both BU index and rainfall, was not able to be run as these variables were correlated. After running the three models (null, BU index, rainfall), we selected the model with the lowest Akaike information criterion (AIC) scores and confirmed that the best model had a ΔAIC value > 2 (Anderson and Burnham 2002). Model weights were also reviewed.

A random forest (RF) classification (Breiman 2001) was run to assess the local environmental variables associated with the risk of *Plasmodium* infection in juncos, specifically focusing on second‐year birds to reduce the chance of including birds with chronic infections (Valkiūnas 2005). This machine learning approach allows us to compare multiple, often correlated variables, without the need for a priori hypotheses, which can help us select the best variables. Our RF was performed via the *ranger* 0.1 package (Wright and Ziegler 2017). The explanatory variables included percent vegetation cover, water cover, human‐associated variables, body morphometrics (body condition, wing chord length, age, and sex), and capture date (ordinal). RF settings were compared to obtain the highest accuracy. The final iteration included the following conditions: 500 trees, mtry = 6, minimum node size = 5, training data based on 80% of samples, and significance determined based on corrected Gini impurity values. The predictor variables were considered significant only if they exceeded the absolute values of the variable with the lowest Gini impurity.

Based on the output from our RF, we prepared a classification tree for all juncos in our study to further assess how the significant variables correlated with infection status. We were specifically interested in determining whether infection status had a nonlinear relationship with the significant variables. To address this, and to consider potential correlation between variables, we ran a classification tree with the *rpart* 4.1 package and visualized it with the *rpart.plot* 3.1 package. By using rpart to run a classification tree, we can produce and review the specific classification rules used to predict infection risks.

### 2.5 Vector Data Collection

The data on vector populations and species identification were provided by the California Vectorborne Disease Surveillance System (CalSurv). CalSurv is a central repository of vector‐borne disease surveillance data collected by the Mosquito and Vector Control Association of California, the California Department of Public Health, and the University of California, Davis. Mosquito abundance data were obtained from 2021, 2022, and 2023 within Los Angeles, Santa Barbara, San Diego, and San Francisco counties. The data were filtered based on the number of traps (*n* = 1), whether problems occurred (FALSE), and whether a species was detected a minimum of 20 times. The final dataset included CO^2^‐baited, CDC, gravid, BG sentinel, New Jersey light, encephalitis vector survey, and unspecified traps. The coordinates for each trap were used to determine the BU index and monthly precipitation as previously described. The filtered dataset was then used to determine how the abundance of each mosquito species responded to urbanization and precipitation. The final, filtered dataset is available online. Initially, two negative binomial GLMMs were run for each species of mosquito, with either (1) cumulative precipitation or (2) the BU index abundance set as fixed variables, the log of abundance set as the response variable, the trap type set as a random intercept, and the number of days a trap was open set as a weighted variable (Carlson et al. 2015). This method proved to be overfit for species where the number of different trap types was ≤ 4. The models were rerun in this case, but the trap type was removed as a random effect. This approach limits our ability to compare variation between species directly but should more accurately reflect how individual species respond to environmental conditions.

## 3 Results

### 3.1 Distribution of Avian Haemosporidian Parasites

**FIGURE 1:**
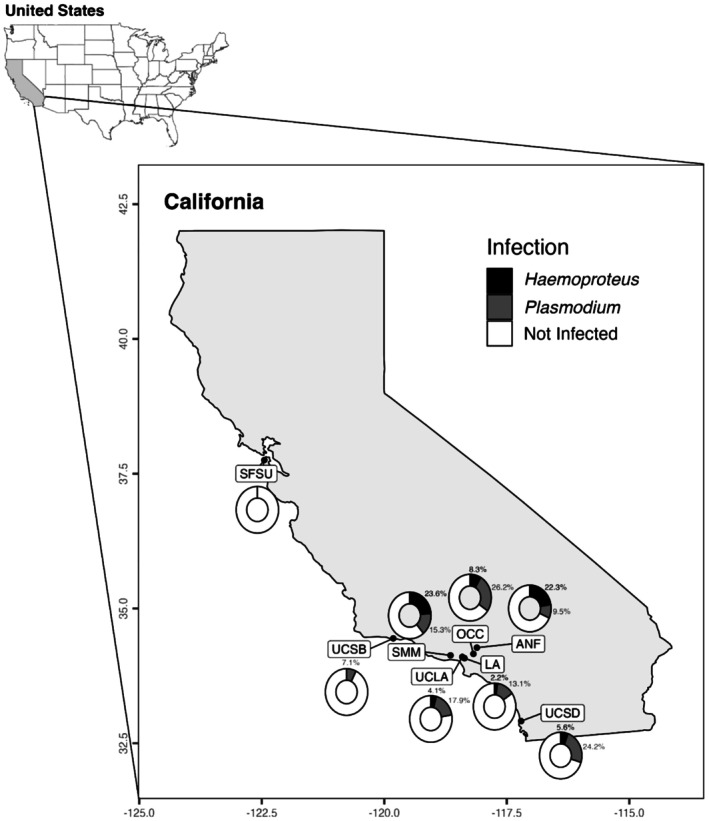
Map of the study sites and the corresponding infection prevalence of *Haemoproteus* (black) and *Plasmodium* (gray) at each site are shown. Mixed infections were not observed and were therefore not included. Sample sites include Angeles National Forest (ANF, *n* = 55), Santa Monica Mountains (SMM, *n* = 18), Los Angeles parks (LA, *n* = 57), Occidental College (OCC, *n* = 20), San Francisco State University (SFSU, *n* = 6), University of California, Los Angeles (UCLA, *n* = 262), University of California, Santa Barbara (UCSB, *n* = 35), and University of California, San Diego (UCSD, *n* = 38).

**FIGURE 2:**
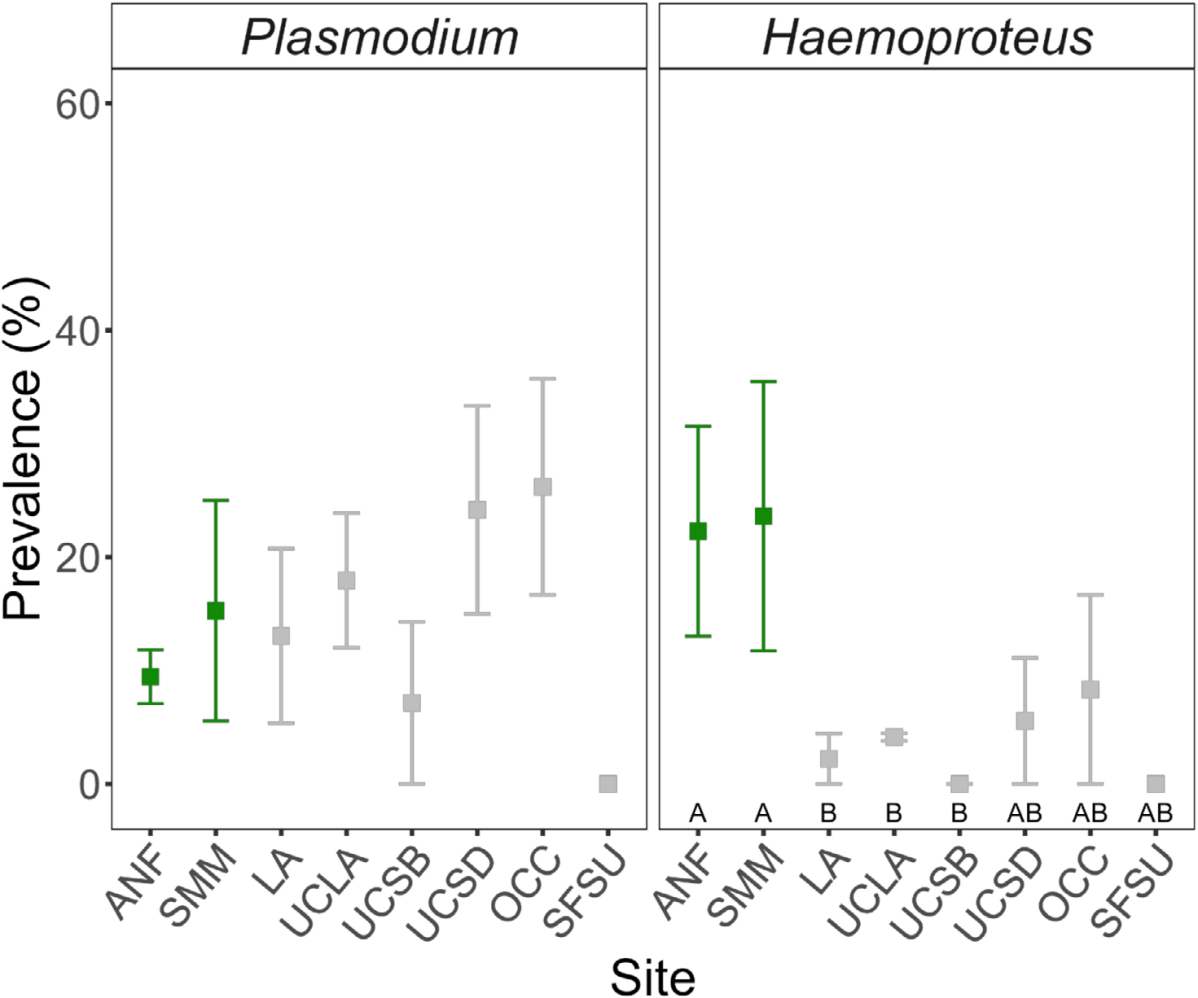
Infection prevalence across study sites. The mean infection prevalence for “non‐urban” (green) and “urban” (gray) sites of both *Plasmodium* and *Haemoproteus* are shown. Bars represent 95% confidence intervals. For *Haemoproteus, l*etters (A and B) represent significance: Same letters indicate no significant differences, and different letters indicate statistically significant differences.

**FIGURE A1:**
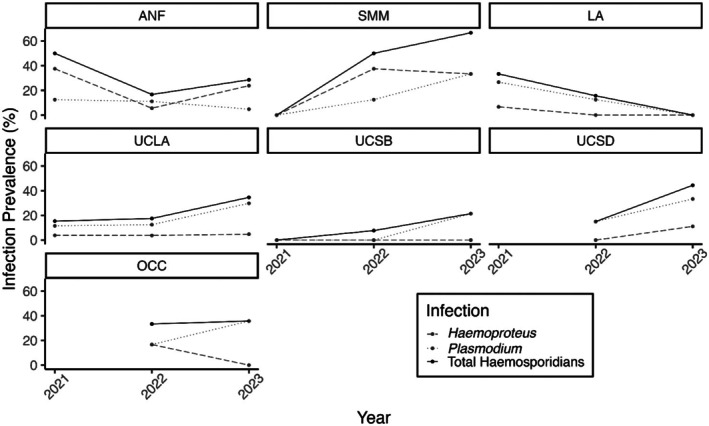
*Haemoproteus* (dashed line), *Plasmodium* (dotted line), and combined haemosporidian infection prevalence (solid line) per site from 2021 to 2023. Sample sites include Angeles National Forest (ANF, *n* = 55), Santa Monica Mountains (SMM, *n* = 18), Los Angeles parks (LA, *n* = 57), Occidental College (OCC, *n* = 20), University of California, Los Angeles (UCLA, *n* = 262), University of California, Santa Barbara (UCSB, *n* = 35), and University of California, San Diego (UCSD, *n* = 38). San Francisco (0% infection prevalence, *n* = 6) was only sampled once (2023) and therefore not included in this figure.

A total of 542 juncos were captured from four metropolitan regions across California over the course of three breeding seasons: 2021 (*n* = 128), 2022 (*n* = 178), and 2023 (*n* = 236). Among these, 28.97% tested positive for haemosporidian parasites, with 15.31% identified as *Plasmodium* infections and 5.72% as *Haemoproteus infections* (Figure 1). The infection prevalence in our study was lower than that reported in a previous study (36%–67%) performed at the same sites in southern California, but a direct comparison cannot be made as those did not differentiate between the two genera (Hanauer 2017). Across all the samples, seven unique lineages of *Plasmodium* (*P. homopolare* BAEBIC02; *lutzi* CATUSI05; spp. TROAED24; *relictum* GRW04; *relictum* JUHEY27; spp. POOHIS04; *cathemerium* SEIAUR01) and six unique lineages of *Haemoproteus* (*H. coatneyi* B00464; spp. CATUST10; spp. GYMSAL01; spp. JUHYE03; spp. JUNHYE28; spp. JUNHYE29) were identified. The population captured at OCC had the highest proportion of birds infected with *Plasmodium* (30%, *n* = 20); however, infections did not significantly differ among sites (Figure 2). In contrast, samples collected from ANF and SMM had the highest proportion of birds infected with *Haemoproteus* (21.82%, *n* = 55; 22.22%, *n* = 18, respectively). The Fisher pairwise analysis (Table 1) revealed that *Haemoproteus* infection was significantly greater in the montane populations of the ANF and SMM than in the LA (Fisher pairwise: *p* = 0.012, 0.049), UCLA (*p* = 0.002, 0.049), and UCSB (*p* = 0.025, 0.049) populations but not in the other urban populations. Infection prevalence per site across years is shown in Figure A1.

**TABLE 1:** Fisher's exact test for pairwise comparisons of the mean incidence of *Haemoproteus. “*Non‐urban” sites include “ANF” and “SMM”, whereas all other sites are in primarily “urban” habitats.

	ANF	LA	OCC	SFSU	SMM	UCLA	UCSB
LA	0.012*	—	—	—	—	—	—
OCC	0.476	0.924	—	—	—	—	—
SFSU	0.924	1.000	1.000	—	—	—	—
SMM	1.000	0.049*	0.476	0.924	—	—	—
UCLA	0.002**	0.981	0.924	1.000	0.049*	—	—
UCSB	0.025*	1.000	0.872	1.000	0.049*	0.870	—
UCSD	0.152	0.924	1.000	1.000	0.269	0.981	0.924

*Note:* The statistical significance of the *p* value is shown: *p < 0.05; **p < 0.001.

**FIGURE A2:**
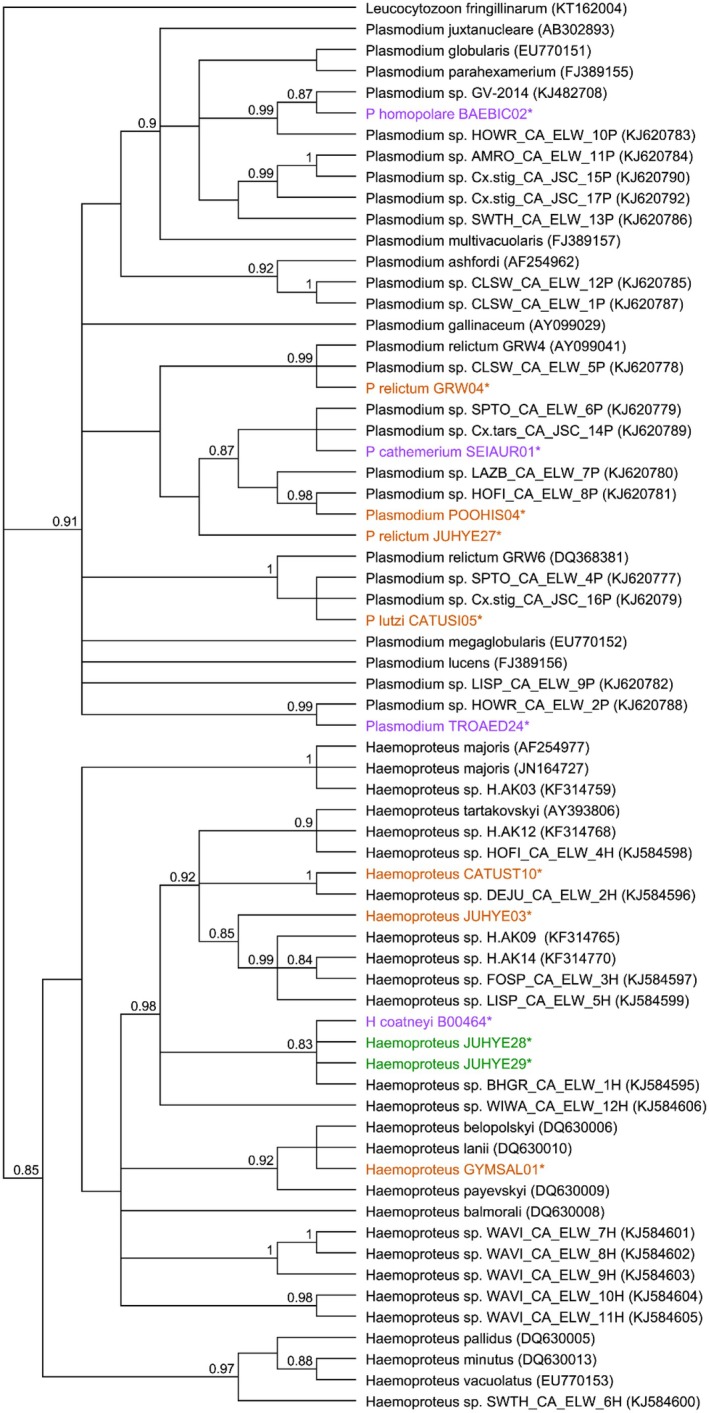
Cladogram of the haemosporidian lineages. Only branches with > 80% posterior probability based on Bayesian inferences are labeled. Reference sequences are shown in black with associated GenBank accession numbers in parentheses. * denotes lineages found in our study. Sequences in orange represent lineages exclusively found in urban habitats, green represents lineages exclusively found in non‐urban habitats, and purple represents lineages found across both habitat types.

Based on our cladogram, the various haemosporidian lineages did not appear to be closely related to each other (Figure A2). However, some *Haemoproteus* lineages (B00464, JUHYE28, JUHYE29) were more closely related to each other than the other reference lineages. Notably, these lineages, along with CATUST10 and JUHYE03, were also shown to form a clade with many other lineages previously reported on the west coast, with many being reported in various sparrow species.

**FIGURE 3:**
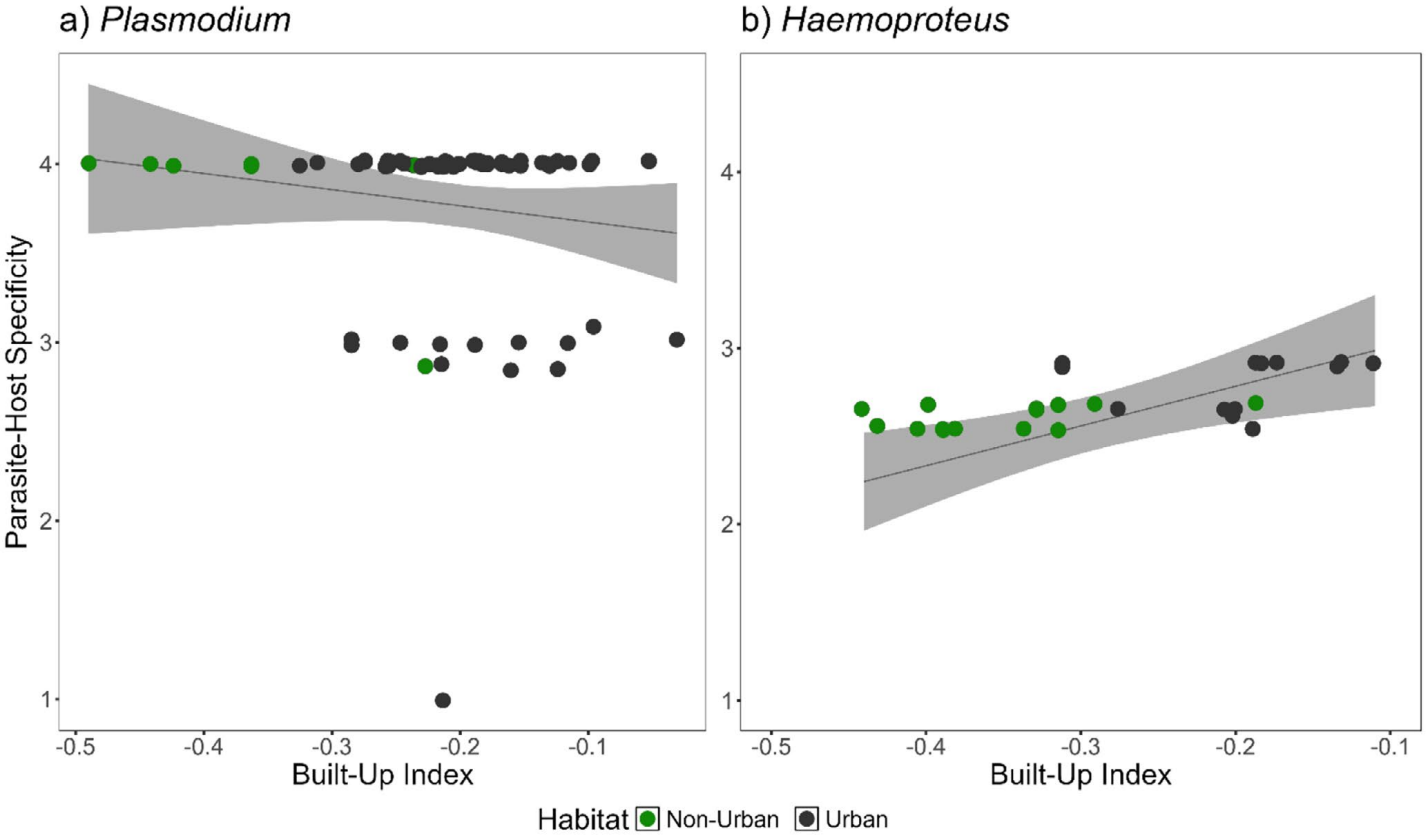
Generalized linear regression with 95% confidence intervals (shaded light gray) of parasite–host specificity (S_TD_) along an urbanization gradient (Built‐Up Index) for (a) *Plasmodium* and (b) *Haemoproteus*. Scatter plot points are jittered along y‐axis for each integer‐value. Low S_TD_ indicates parasites have only been reported in closely related species. Color differences, “non‐urban” sites in green and “urban” sites in black, are provided for improved visualization but were not included as variables in model.

Host–parasite specificity for both genera of parasites was compared to assess whether lineages presented reduced levels of host specificity as urbanization increased. *Plasmodium* lineages primarily consisted of host–parasite specificity values of ~4 and did not significantly vary in response to urbanization (Figure 3a; GLM, β = 0.06 ± 0.05, *p* = 0.21). In contrast, *Haemoproteus* lineages showed higher variability of host specificity and were significantly more generalist in urban habitats (Figure 3b; GLM, β = 0.334 ± 0.12, *p* = 0.012*).

**FIGURE 4:**
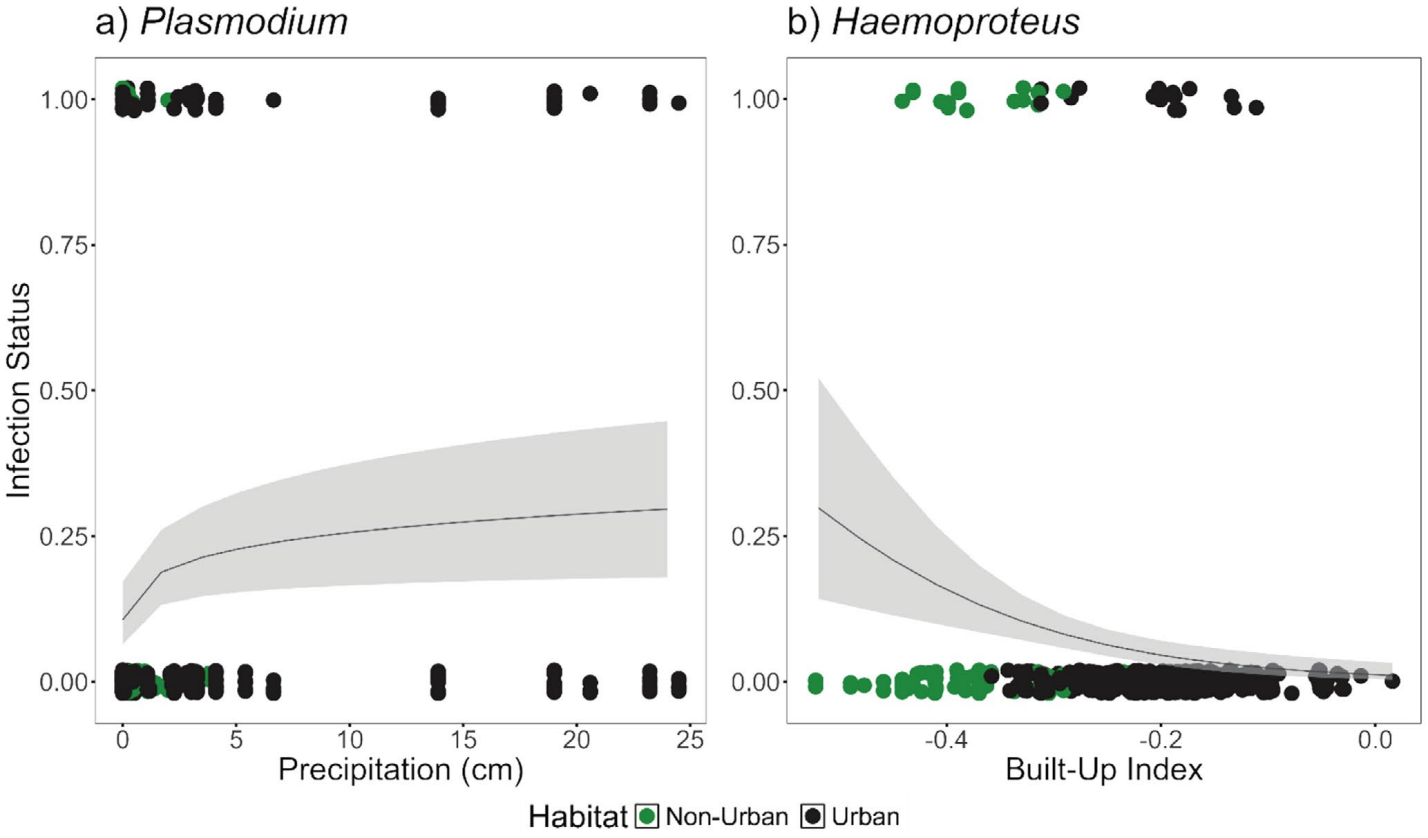
Plotted generalized linear mixed effect model and 95% confidence intervals (shaded light gray) for (a) *Plasmodium* and (b) *Haemoproteus*. Points are jittered along the y‐axis. Color differences, “non‐urban” sites in green and “urban” sites in black, are provided for improved visualization but were not included as variables in model.

To assess the role of broad environmental variation, either urbanization or precipitation, a set of GLMMs was run comparing the infection status of birds for either genera of parasites. For juncos infected with *Plasmodium*, the best‐fit model had infection status as a response to monthly rainfall (Figure 4; Precipitation: β = 0.23 ± 0.08, *z* = 2.80, *p* = 0.005*; Table 2).

**TABLE 2:** Top generalized linear mixed effect models for infection status of *Plasmodium* (AIC: 441.1; Weight: 91.4%) and *Haemoproteus* (AIC: 223.5; Weight: 99.5%).

	Variable		Estimate	Std error	*z* value	Probability
*Plasmodium*	Model	Intercept	−2.128	0.284	−7.485	< 0.001***
		log(Precipitation)	0.230	0.082	2.796	0.01*
	Random effect	Month				
*Haemoproteus*	Model	Intercept	−4.411	0.545	−8.094	< 0.001***
		BU Index	−6.839	1.834	−3.728	< 0.001***
	Random effect	Month				

*Note:* The infection status of birds for the specified genus of parasite was set as the response variable. The explanatory variables are either the log of the cumulative monthly precipitation of the capture date (cm) or the degree of urbanization measured by Built‐Up Index. Month of capture was set as a random effect to account for possible seasonal variation in vector abundance. *p*‐values are included: *p < 0.05; **p < 0.01; ***p < 0.001.

In highly urban habitats, *Haemoproteus*‐infected juncos were nearly absent. This is supported by the best‐fit model for *Haemoproteus* infections. Degree of urbanization was negatively correlated with infection status (Figure 4; BU, β = −6.84 ± 1.83, *z* = −3.73, *p* < 0.001***; Table 2).

**FIGURE 5:**
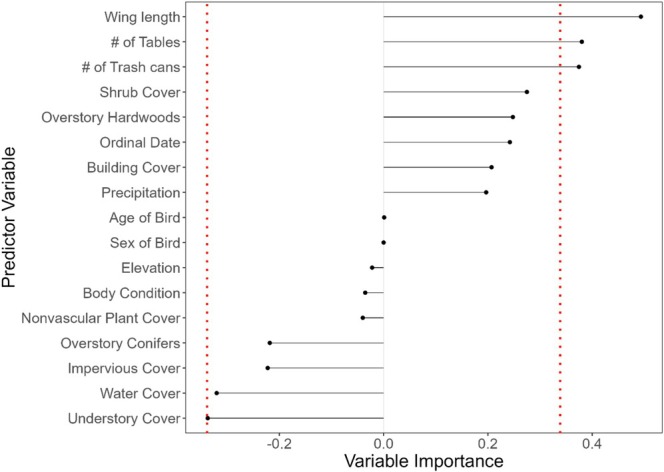
Random Forest classification of local habitat characteristics within 50 m of a capture site and host morphological characteristics as predictors of presence/absence of *Plasmodium* infections. Variable importance was calculated via the corrected Gini impurity index. Significance was set at the absolute value of the lowest Gini impurity index value (dotted red line). Variables that exceeded this threshold were considered significant.

A random forest classification was run to assess which local habitat conditions were most predictive of whether a junco would likely acquire a *Plasmodium* infection in a particular habitat. As none of the sites had significantly different infection prevalence, all the sites were included in the random forest. Across the various iterations of the random forest, the maximum accuracy was 84.7%. The understory cover was found to have the lowest importance value at −0.337, and the cutoff was set to the absolute value (−0.337 to 0.337, Figure 5). Using this cutoff value, three variables—wing chord length (mm) (importance = 0.494), the number of trash cans present in a habitat (importance = 0.38), and the number of tables (importance = 0.375)—exceeded this threshold and were considered significant.

**FIGURE 6:**
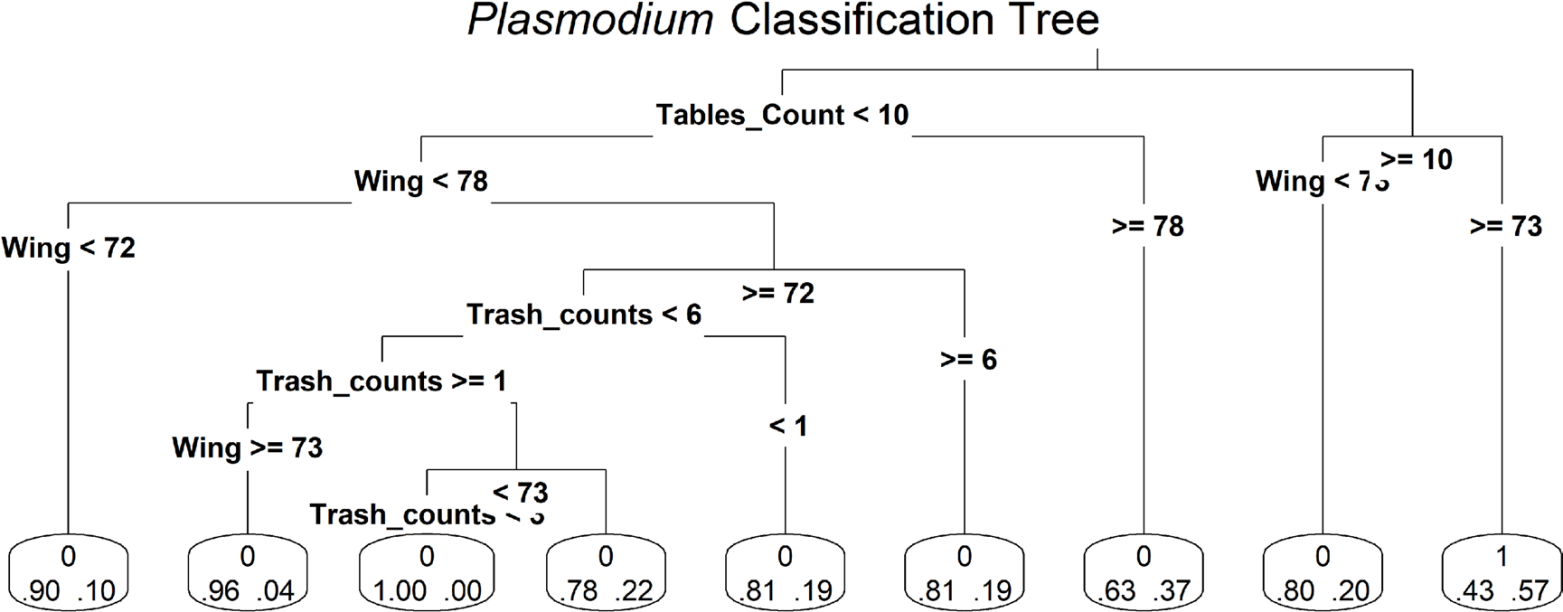
Classification tree of *Plasmodium*‐infected second‐year dark‐eyed juncos. Variables were set on the basis of significance from Random Forest classification. Individuals were classified as infected (1) or not infected (0) using a threshold set to > 50% chance. At each final leaf, the proportion of samples that met the decision criteria and were not infected is shown on the bottom left, and the proportion that were infected in *Plasmodium* are shown on the bottom right. The table count and trash count refers to absolute values, “Wing” refers to wing chord length (mm).

Data from all individuals were used to run a classification tree to assess further how wing chord and human activity related to infection prevalence. We found that the risk of infection was highest in areas with many tables (> 10) and for birds with relatively long wings (≥ 73 mm). Infected birds were still identified under different habitat conditions, with the exception of birds with relatively short wings (72–73 mm) whose territories had few tables (< 10) and trash cans (1–3) (Figure 6). Birds that met these criteria showed no instances of *Plasmodium* infections.

**FIGURE A3:**
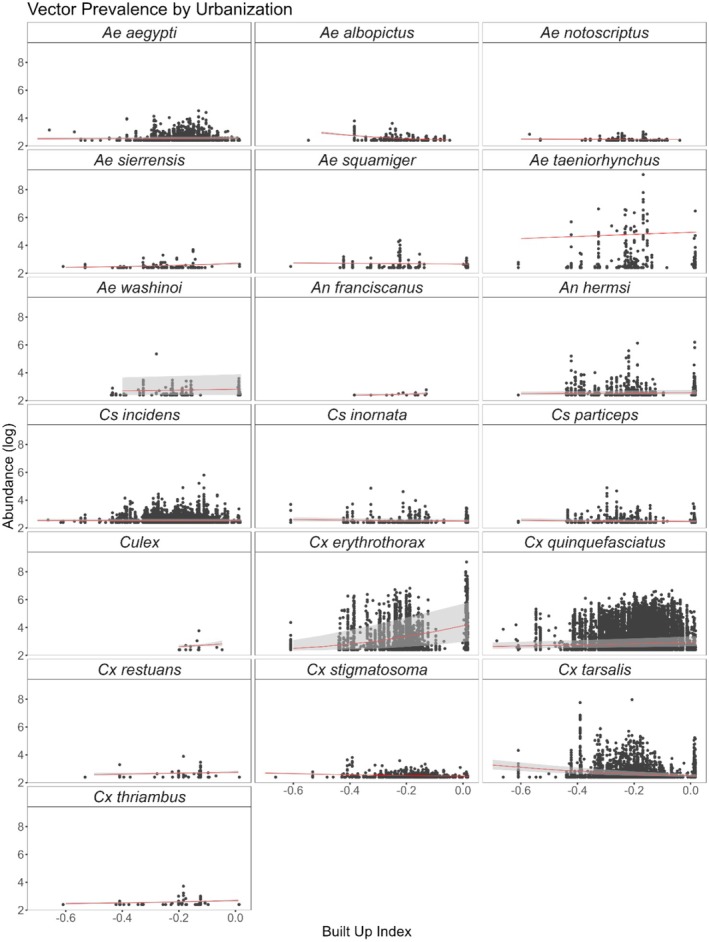
Generalized linear (mixed) models of the abundance of each vector species from 2021 to 2023 in response to the degree of urbanization (Built‐Up Index) with higher values indicating more urban habitat. The gray dots represent the log of the abundance. The red lines show the predicted trendline based on the appropriate model. The samples were sorted by species when possible and grouped by genus when species‐level identification was not confirmed.

**FIGURE A4:**
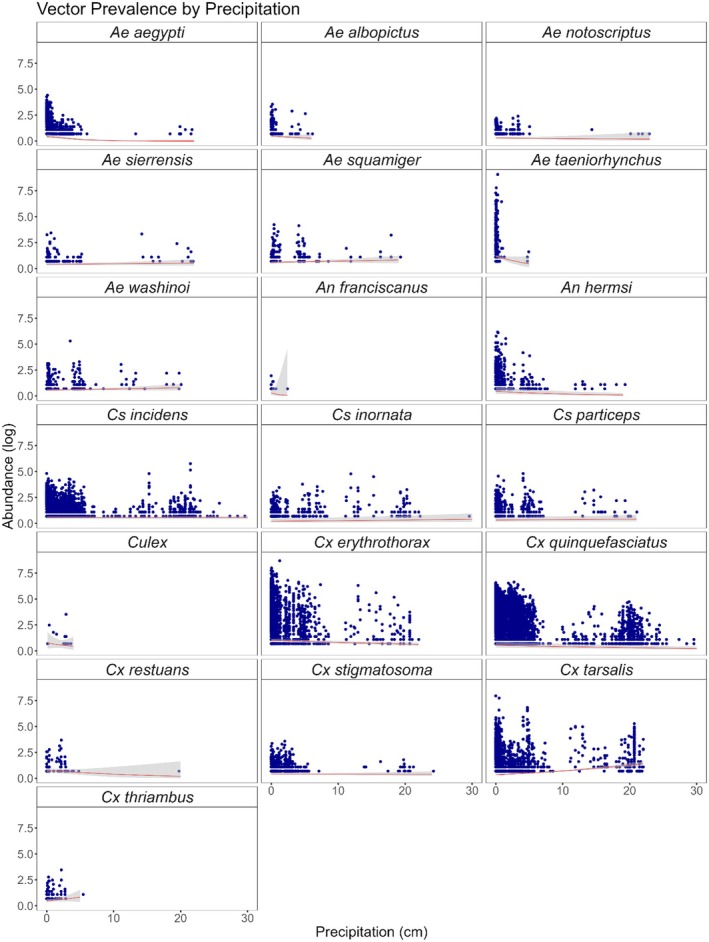
Generalized linear (mixed) models of abundance for each species of vector from 2021 to 2023 in response to cumulative monthly precipitation averaged 50 m around each trap site. The blue dots represent the log of abundance. The red lines show the predicted trendline based on the appropriate model. The samples were sorted by species when possible and grouped by genus when species‐level identification was not confirmed.

Nineteen species of mosquitoes (*n* = 618,871) were observed across the study sites. Not accounting for sampling effort, *Cx quinquefasciatus* was the most abundant (*n* = 23,917), followed by *Cs incidens* (*n* = 7109), and *Cx tarsalis* (*n* = 4789). The least abundant species were *An franciscanus* (*n* = 37) and *Cx restuans* (*n* = 76). Responses for each species to urbanization (Figure A3) or precipitation‐based (Figure A4) are provided based on the appropriate model GLM or GLMM with specific trends provided (Table A1). Only *Cx tarsalis* abundance increased in response to both precipitation and urbanization.

## 4 Discussion

Urbanization is a strong selective force that has altered organismal traits and community composition (Blair 2001; Yeh and Price 2004; McKinney 2006; Aronson et al. 2014; Callaghan et al. 2019; Friis, Atwell, et al. 2022; Neate‐Clegg et al. 2023; Diamant and Yeh 2024). Although the impacts of urbanization on many charismatic macroorganisms have been investigated, cryptic species or microorganisms can prove more challenging to study (Magle et al. 2019). These limitations can make understanding more complex ecological interactions difficult, especially in the case of intracellular obligate parasites. Our study uses a parasite system, that of avian haemosporidians, to further our understanding of the connection between multiscale environmental changes associated with urbanization and the diversity and prevalence of haemosporidians across several junco populations throughout California.

Urbanization can have a homogenization effect on parasites, resulting in more generalist parasites. This is seen in the lineages of *Haemoproteus* parasites, which were found to be primarily specialists but were more generalist as urbanization increased (Figure 3). *Haemoproteus* spp. GYMSAL01 (STD= 2.91) was the most generalist of the *Haemoproteus* lineages and was exclusively found in urban habitats. Previous studies further support this generalist life history; details on the additional lineages are available online (Oakgrove et al. 2014; Martínez‐Renau et al. 2022). This generalist life history suggests parasites in urban habitats should be capable of transmitting to a larger number of host species compared to those in non‐urban habitats and is supported by data on vector communities in urban habitats (Santiago‐Alarcon et al. 2013).

Although additional studies are necessary, we provide insight regarding how birds are acquiring *Haemoproteus* infections. Based on long‐term observation data (Walters et al. 2023; Diamant and Yeh 2024), juncos have high site fidelity within and between years during the breeding season. This conclusion is supported by a study on homing success in migrant and non‐migrant juncos (Keiser et al. 2005), but a formal study would be necessary to verify these observations. With these limitations in mind, adult males have been continuously observed at their specific territories throughout the breeding season and between years. Adult female juncos show lower fidelity, occasionally switching to a different male following a nest failure. One female was spotted 2 km from the initial capture site 1 year after being captured. Juveniles travel significantly farther after their first year, with the current maximum reported distance of 9 km. The wintering habitats and migration routes of our non‐urban populations are currently understudied. Wintering birds have been shown to acquire infections (Soares et al. 2020) which could contribute to differences in infection status among birds. However, based on our results, if wintering habitats and differences in migration patterns are significantly contributing to haemosporidian infections, *Haemoproteus* is more likely to be impacted than *Plasmodium*. This is because our results show that despite the non‐urban dark‐eyed junco populations being migratory and the urban populations being primarily year‐round residents (Yeh and Price 2004; Bressler et al. 2020; Diamant and Yeh 2024), there were no differences in *Plasmodium* infections. In contrast, more birds were infected with *Haemoproteus* parasites in non‐urban sites. This raises an important question about whether non‐urban juncos are becoming infected with *Haemoproteus* locally or in their wintering habitats. Future studies on biting midge populations within cities or sampling from the non‐urban wintering habitats would allow us to assess whether *Haemoproteus* infections are being locally extirpated due to urbanization or the product of infections following migration to wintering habitats.

*Plasmodium* infection status did not vary in response to landscape variation, based on the degree of urbanization, but was positively correlated with increased precipitation (Figure 4 and Table 2). A connection between water availability, whether in the form of precipitation or bodies of water, and *Plasmodium* has been previously reported (Beadell et al. 2004; Bonneaud et al. 2009; Sebaio et al. 2010; Sehgal et al. 2011; Gonzalez‐Quevedo et al. 2014; Carbó‐Ramírez et al. 2017; Ferraguti et al. 2018; Tchoumbou et al. 2020; Menzies et al. 2021; De Angeli Dutra et al. 2023) and is an important consideration for studies examining the effects of infections on hosts. Water availability may be especially important within areas that experience prolonged drought, as is the case for much of California. Therefore, studies that require larger sample sizes to examine the effects of *Plasmodium* on wild birds could require higher‐intensity field work and/or may need to be postponed until conditions become favorable.

Although broad land use changes associated with urbanization did not correspond with changes to *Plasmodium* infection status, we were interested in determining which, if any, localized habitat conditions could contribute to increased infection risk. Our results found that wing chord length and human activity levels, based on the number of trash cans and tables, were the most significant variables for predicting infection risk of second‐year birds (Figure 6). These results suggest that biotic factors—rather than abiotic environmental conditions—may play a more significant role in increasing infection risk for young juncos. However, while our RF model achieved a maximum accuracy of 86%, further studies are needed to determine whether these patterns hold consistently across different sites and datasets.

With these limitations in mind, we were interested in examining how these variables corresponded to infections across our study sites. Based on our classification tree, juncos with long wings that established territories with substantial human presence were the most likely to be infected with *Plasmodium*. Longer wing chord, stated to be a metric of larger body mass, was previously observed to correspond with infection status in house sparrows ( *Passer domesticus* ) (Jiménez‐Peñuela et al. 2019). However, based on our RF, body condition, based on the linear regression between body mass and tarsus length, did not significantly increase infection risk. Further, for dark‐eyed juncos, tarsus length appears to remain consistent between populations, but wing chord length was found to differ across populations (Diamant and Yeh 2024). Based on this, differences in wing chord length could be reflective of increased risk of infection due to differences in behaviors, rather than differences based on body size. For instance, while longer wing chords contribute to increased flying distance, there is reduced maneuverability (Tittler et al. 2009) leading to increased risk of predation by mosquitoes. Similarly, some defensive behaviors depend on wing movement (Darbro and Harrington 2007) which may also be reduced with long wing length. An increase in human presence would also increase the presence of mosquitoes. Human activity can increase food and resource availability, becoming an attractive habitat for human‐tolerant birds and mosquito species leading to increased transmission (Densmore and French 2005; Sol et al. 2017; Brown et al. 2022; Mazué et al. 2022; García‐Arroyo et al. 2023; Arsenault‐Benoit and Fritz 2023). Together, our RF and subsequent classification tree suggest that biotic characteristics, rather than abiotic or environmental conditions of a bird's specific territory, play a larger role in the risk of infection, likely due to increased risk of encountering a potential vector.

Given the potentially important role of mosquito vectors in sustaining *Plasmodium* infections, we aimed to identify which mosquito species might serve as potential vectors. Exact parallels between the response of mosquito abundance and avian infection prevalence to urbanization and precipitation were not observed, limiting our ability to determine potential urban *Plasmodium* vector species, but *Cx tarsalis* is notable for being the only species to increase with precipitation. *Cx tarsalis* is also known to be a competent vector of *Plasmodium* (Carlson et al. 2015) as well as some zoonotic pathogens, such as West Nile virus (Kent et al. 2009). Considering the increase in *Cx tarsalis* within urban habitats found in our study and the seasonal differences in feeding preferences (Kent et al. 2009), it would be beneficial for both human and wildlife health to examine the role of *Cx tarsalis* in avian pathogen transmission.

### 4.1 Limitations

There are important limitations in our study but may prove fruitful for future studies. Firstly, host specificity was calculated based on data currently available in the MalAvi database (Bensch et al. 2009). Although a significant resource, this data is limited by sampling efforts and could result in potential false negatives and underestimating parasite–host specificity. A few methodological limitations could have also affected the accuracy of our results. Infected birds have shown reduced activity levels compared to non‐infected birds, and mist netting can underestimate naturally occurring infections in free‐living birds (Valkiūnas 2005). When infected birds are captured, the nPCR approach may also affect detectability due to a bias towards *Haemoproteus* over *Plasmodium* (Ciloglu et al. 2019). To account for this, future studies may benefit from using genus‐specific primers that have since been developed (Musa et al. 2024). Considering the relatively small difference between different lineages (1% difference), having high‐quality sequences is necessary (Bensch et al. 2009). To account for this, we limited our results to samples with > 80% high‐quality sequence. Additionally, the analysis of local vegetation is limited by our current understanding of junco movement. Based on our long‐term observational data and a few related studies (Chandler et al. 1994; Keiser et al. 2005), juncos are expected to establish 50 m territories within and between years. Formal studies could assist in verifying these assumptions and would be necessary to assess where birds *acquire* infections, as opposed to our study that can only report on the presence/absence of infections. Lastly, while we expect vector communities to correspond with the transmission and prevalence of *Plasmodium* infections, the available data did not directly correspond with junco‐capture sites. This limits our interpretation to population‐level patterns, but future studies, especially in collaboration with vector control agencies, could explore site‐specific vector‐host–parasite distribution.

## 5 Conclusions

Urbanization is an important force that is rapidly changing environments. These changes are not uniform and can have significantly variable consequences for different species. In this study, we examined how urbanization at different scales corresponded with infection prevalence of host‐specialist, *Haemoproteus*, and host‐generalist, *Plasmodium*, parasites. On a broad scale, urbanization was negatively correlated with host‐specialist parasites, suggesting a potential loss of biodiversity. While local habitats can have a moderate role in shaping which individuals are at risk of infection, broad‐scale variables, specifically precipitation, are likely to have a stronger role. As urbanization continues to drive homogenization and generalist life histories, it is becoming increasingly important to understand the impacts on disease dynamics, including the effects on local vectors, to support human, wildlife, and ecosystem health.

## Author Contributions

**Wilmer Stanley Amaya‐Mejia:** conceptualization (lead), data curation (lead), formal analysis (lead), funding acquisition (lead), investigation (lead), methodology (lead), project administration (lead), writing – original draft (lead). **Lillian Ma:** data curation (supporting), formal analysis (supporting), writing – original draft (supporting). **Sara Freimuth:** data curation (supporting), methodology (supporting), writing – original draft (supporting). **Ravinder N. M. Sehgal:** conceptualization (supporting), methodology (supporting), supervision (equal), writing – original draft (supporting). **Pamela Yeh:** conceptualization (supporting), funding acquisition (equal), resources (lead), supervision (equal), writing – review and editing (equal).

## Ethics Statement

All animal handling in this study adhered to protocols approved by the Institutional Animal Care and Use Committee (IACUC) of UCLA (ARC‐2018‐007‐AM‐004). Banding efforts were conducted in compliance with the Ethics and Responsibilities of Bird Banders published by the US Geological Survey Federal Bird Banding Laboratory (Permit #23809) and as outlined by the State of California Department of Fish and Wildlife Scientific Collecting Permit—Specific Use (S‐191300002‐20,288‐001‐02) for taking/possession of wildlife for scientific purposes. This study follows all relevant ARRIVE methods required for observational animal research.

## Conflicts of Interest

The authors declare no conflicts of interest.

## Data Availability

Data is available in text and online or by contacting the authors. Online data is available: https://doi.org/10.5061/dryad.rr4xgxdj0.

## Appendix A

**TABLE A1:** Vector responses to precipitation (PPT) and urbanization (BU).

Species	Traps	Model	PPT estimate	*p*	BU Estimate	*p*	Previous study
Ae aegypti	6	GLMM	−0.064983	< 0.001***	0.6485	0.099	
Ae albopictus	3	GLM	−0.01363	0.42	−3.2946	< 0.001***	
Ae notoscriptus	4	GLM	0.00419	0.199	0.8953	0.436	
Ae sierrensis	3	GLM	0.01834	0.164	1.4567	0.279	*
Ae squamiger	3	GLM	−0.00291	0.326	−0.2972	0.571	
Ae taeniorhynchus	4	GLM	−0.06897	0.027*	0.02926	0.95	
Ae washinoi	5	GLMM	−0.003463	0.279	0.7839	0.134	*
An franciscanus	3	GLM	−0.00185	0.997	14.292	0.171	
An hermsi	6	GLMM	−0.010253	0.007**	−0.1432	0.643	
Cs incidens	6	GLMM	−0.0008816	0.148	−0.4279	0.011*	*
Cs inornata	6	GLMM	0.002292	0.083	0.07179	0.911	*
**Cs particeps**	6	GLMM	0.00001481	0.099	−0.3391	0.322	*
Culex	2	GLM	0.01226	0.807	5.2833	0.545	
Cx erythrothorax	7	GLMM	−0.0056173	< 0.001***	1.1295	< 0.001***	*
**Cx quinquefasciatus**	10	GLMM	−0.0080167	< 0.001***	0.68295	< 0.001***	*
**Cx restuans**	3	GLM	0.02525	0.306	1.7693	0.227	*
**Cx stigmatosoma**	3	GLM	−0.028533	< 0.001***	−0.6449	0.152	*
**Cx tarsalis**	8	GLMM	0.016978	< 0.001***	1.77823	< 0.001***	*
Cx thriambus	3	GLM	0.01525	0.3299	2.24158	0.106	*

*Note:* Vector abundance was set as the response variable. Species name, β‐values, and *p*‐values are all included. Traps refer to the number of different trap types used to capture that species across all sites. Models refer to generalized linear model (GLM), if less than 5 different trap types were used, or generalized linear mixed models (GLMM) when more than 5different trap types were used, with the number of traps set as a random effect. Highlighted colors reflect trends: red = decrease in response to rainfall, blue = increase in response to rainfall, gray = increase in response to urbanization, green = decrease in response to urbanization. Previous study refers to Carlson et al. 2015 and denotes that the mosquito species was captured (*) and bolded species indicate these were capable of being a competent *Plasmodium* vector.
